# Solid fuel smoke exposure and risk of obstructive airways disease

**DOI:** 10.1186/1735-2746-9-8

**Published:** 2012-10-18

**Authors:** Mostafa Qorbani, Masud Yunesian

**Affiliations:** 1 Department of Community Medicine, Golestan University of Medical Sciences, Gorgan, Iran; 2 Department of Epidemiology and Biostatistics, School of Public Health, Tehran University of Medical Sciences, Tehran, Iran; 3 Department of Environmental Health Engineering, School of Public Health, Tehran University of Medical Sciences, Tehran, Iran; 4 Center for Air Pollution Research, Tehran University of Medical Sciences, Tehran, Iran

**Keywords:** Obstructive airway disease, Indoor air pollution, Smoke, Baking home-made bread

## Abstract

This study was designed to investigate whether there is an association between Obstructive Airways Disease (OAD) and indoor exposure to baking home-made bread smoke (BHBS) in ground oven at home. In this hospital-based case–control study, 83 patients with OAD (cases) were compared with 72 patients without any known pulmonary diseases from the surgical ward (controls) who were frequently matched with cases on age. The interview was performed using the modified questionnaire recommended by the "American Thoracic Society". The questionnaire comprised of demographic information, occupational history, cigarette smoking and indoor exposure to BHBS in ground oven at home. The exposure to BHBS was considered both as a dichotomous and quantitative variable (number of years being exposed to smoke) and the population attributable fraction (PAF) was estimated due to BHBS exposure. The percentage of indoor exposure to BHBS was measured as 51.8% and 30.6% in the cases and the controls, respectively. The average years of exposure to BHBS was 20.46 years (SD: 11.60) for the cases and 15.38 years (SD: 13.20) for the controls. The univariate analysis comparing the cases and the controls showed that exposure to BHBS (as a binary variable) and occupational exposure to dust was significantly associated with OAD. In the multivariate model, only exposure to BHBS was associated with OAD (OR=2.22, 95%CI = 1.14-4.35). Duration of exposure to BHBS (as a quantitative variable) was significantly associated with OAD in the univariate model. In the multivariate model, only the duration of exposure to BHBS (years) showed a significant association with OAD (OR=1.04, 95% CI=1.01-1.08). Population attributable risk due to BHBS exposure was equal to 28.5%.

## Introduction

Indoor Air Pollution (IAP) is one of the major health problems in the developing countries like Iran that is resulted from incomplete combustion of the solid fuels such as wood, animal dung, agricultural crop residues and coal. Incomplete combustion of the solid fuels in the domestic traditional stoves in the developing countries produces a lot of carbon monoxide, nitrogen free radicals, hydrocarbons and hazardous pollutants. It is estimated that about half of the world's countries and more than 3 billion people in the world use solid fuels for cooking; most of these countries are located in Asia, Africa and South America [1,2]. In many of these countries, about 90% of the rural households use solid fuels as the main fuel for food preparation, baking and home heating [3]. It is estimated that in the year 2000, approximately 2 million deaths in the world have been attributable to IAP, which is about 5% of the whole world's deaths; the major causes of death due to IAP are the lower respiratory infections (LRI) and chronic obstructive pulmonary disease (COPD) [4,5].

Several studies have emphasized on the role of IAP as one of the etiologies of some respiratory diseases such as LRI, lung cancer, tuberculosis, asthma and COPD [6-10]. In the rural areas of Iran, solid fuels and wood are widely used for baking home-made bread in the ground ovens; this is even common in the winter within closed places with inadequate ventilation, which increases the amount of exposure to smoke. Duration and frequency of baking varies depending on the household size from daily to once a week, while the baking process takes 2–3 hours each time.

Obstructive airway disease (OAD) is the fourth cause of death with about three million attributable deaths worldwide; about 90% of the deaths due to OAD occur within the low and medium income countries [11] Although previous studies have reported age, sex, cigarette smoking, occupational dust exposure, air pollution and indoor smoke exposure as the risk factors of the disease, however, the limited case-report studies conducted in Iran indicate that most of the cases of OAD are presented among the nonsmokers with no history of occupational exposure to smoke who are living in non-polluted areas; most of these people reported exposure to smoke during baking home-made bread in ground ovens in the distant years [12]. With regard to the methodology of previous studies, a robust case–control study was designed to seek and quantify any possible relationship between OAD and indoor exposure to BHBS.

## Materials and methods

This hospital-based case–control study was conducted during September 2009 to December 2010 in the Imam Khomeini Hospital, Tehran. The cases were selected from the pulmonary ward and the controls from the surgical ward of the hospital. 83 patients with OAD were considered as the cases and 72 patients without any known pulmonary diseases referred to the surgical ward were selected as the control group. Case definition was according to clinical diagnosis of OAD, FEV_1_/ FVC less than 70%, FEV_1_ less than 70% of expected predicted value and without clinical history of asthma and control diagnosis was confirmed according to medical history and chest CT. The data collection tool in this study was the "American thoracic society questionnaire for epidemiologic studies" [13]. The questionnaire comprised of demographic information, family history, past medical and occupational history and, cigarette smoking. In particular, some questions were added regarding information on variables related to indoor exposure to BHBS in ground ovens at home, the onset, the duration of exposure and for the frequency of exposure per week. Cigarette smoking exposure was estimated in pack-years. A pack was defined as 20 cigarettes.

Frequency matching was used for the variable of age; for each 10 cases, 10 controls with the same age range were selected (age ranges: 20–35, 35–49, 50–69 and ≥70 years). The interviews were conducted by two trained interviewers when the patients were admitted to the ward. In this study, BHBS's exposure frequency, duration and the onset time of exposure were the main independent variables which were considered both as a dichotomous variables (exposure positive / exposure negative) and a quantitative (number of years of exposure) variable; if the subject reported no exposure, the years of exposure was considered zero and if the individual was exposed, the number of years of exposure was considered accordingly. Cigarette smoking exposure was estimated in pack-years and education was quantitatively considered as the years of successful education.

The data analysis was performed using SPSS version 16. To investigate the factors affecting OAD development, logistic regression model was used to measure the crude odds ratio (OR) of developing OAD for each of the independent variables. Multiple logistic regression (MLR) was used to control the confounders and interactions; the independent variables with a p <0.20 in the univariate analysis were entered in the MLR model. The relationship between indoor exposure to BHBS with OAD was investigated as a trend function (exposure duration and dose) using a Chi square test for trend. The results are expressed as OR with 95% confidence interval (CI). The significance level was adjusted as <0.05.

Population attributable fraction (PAF) due to BHBS exposure was estimated in the study population according to the formula:

(1) $$\left\{PAF=\frac{Pc\left(OR-1\right)}{OR}\right\}$$

In this formula, adjusted odds ratio (OR _adjusted_) was used as a proxy for adjusted relative risk (RR _adjusted_), and P_C_ as proportion of cases exposed to risk factor [14].

## Results

The mean age of the cases and the controls was 52.20 (SD: 16.16) and 52.75 (SD: 16.60) years respectively. The mean cigarette smoking was 21.74±12.53 pack-year in the cases and 18.91±8.07 pack-year in the controls while the males consisted 56.6% of the cases (47 patients) and 59.7% of the controls (43 patients). 51.8% of the cases and 30.6% of the controls reported history of indoor exposure to BHBS. The distribution of baseline characteristics by cases and controls is shown in Table 1. Ethnicity of 14 individuals was unknown.

**Table 1:** Distribution of the baseline characteristics of the subjects referred to Imam Khomeini Hospital, Tehran, separately for the two groups

**Variable**		**Cases (n=83) Number (%)**	**Controls (n=72) Number (%)**
Marital status			
	Single	7 (8.4)	10 (13.9)
	Married	72 (86.7)	62 (86.1)
	Widowed/divorced	4 (4.8)	0 (0)
Ethnicity			
	Fars	35 (45.5)	26 (40.6)
	Turk	25 (32.5)	26 (40.6)
	Kurd	5 (6.4)	3 (4.4)
	Others	12 (15.6)	9 (9)
Gender			
	Male	55 (66.3)	43 (59.7)
	Female	28 (33.7)	29 (40.3)
Cigarette smoking			
	Yes	36 (43.4)	24 (33.3)
	No	47 (56.6)	48 (66.6)
Duration of cigarette smoking (years)		29.34 (13.80)	30.43 (15.34)
Age at cigarette smoking onset (years)		21.33 (6.17)	20.91 (7.85)
Education (years)		6.03 (5.40)	5.8 (5.31)
Age (years)		52.20 (16.16)	52.75 (16.60)

The data for duration of cigarette smoking, age at cigarette smoking onset, education and age are expressed as mean (SD).

When indoor exposure to BHBS was considered as a dichotomous variable, the indoor exposure to BHBS, occupational exposure to dust and cigarette smoking showed P-values less than 0.2 in the univariate logistic regression model. Stratified analysis indicated that the relationship between indoor exposure to BHBS and OAD remained unchanged in the layers of stratified variables (cigarette smoking status, sex, age group, occupational exposure to dust and education). The interaction test (homogeneity) showed no significant interaction. The MLR model showed that only indoor exposure to BHBS was statistically significant (OR: 2.22, CI 95%: 1.14-4.35) (Table 2).

**Table 2:** The relationship between the independent variables and obstructive airway disease in the study subjects

**Variable**	**Cases (%) (n=83)**	**Controls (%) (n=72)**	**Crude OR (95% CI)**	Adjusted OR^**^(95% CI)
BHBS (Y/N)	43 (51.8)	22 (30.6)	2.44 (1.26-4.73)	2.22 (1.14-4.35)
Gender (F/M)	28 (33.7)	29 (40.3)	0.75 (0.39-1.45)	
Occupational exposure to dust(Y/N)	42 (50.6)	23 (31.9)	2.18 (1.13-4.02)	1.46 (0.68-3.10)
Cigarette smoking (pack-year)^*^	12.53 (21.74)	8.07 (18.91)	1.01 (0.99-1.02)	1.00 (0.99-1.02)
Education (years)^*^	6.03 (5.40)	5.8 (5.31)	1.00 (0.95-1.07)	

^*^The data for cigarette smoking and education are expressed as mean (SD).

^**^ Adjusted for BHBS, occupational exposure to dust and cigarette smoking using MLR.

When indoor exposure to BHBS was considered as a quantitative variable (years of exposure), the indoor exposure to BHBS, occupational exposure to dust and cigarette smoking had P-values less than 0.2 in the univariate logistic regression model; the crude odds ratio of developing OAD for the duration of indoor exposure to BHBS (years) was 1.05 (95% CI: 1.02-1.08). After adjusting for the confounders, the MLR model indicated that only indoor exposure to BHBS (years) was statistically significant (OR: 1.04, 95% CI: 1.01–1.08).

The relationship between indoor exposure to BHBS and developing OAD was also clear in the trend analysis. The Table 3 shows that the years of indoor exposure to BHBS and its frequency per week was significantly more in the case group compared with the control group. Besides, the increase in the years of indoor exposure to BHBS and the frequency of exposure increases the risk of developing OAD (p_χ2 for trend_ <0.01)

**Table 3:** The relationship between smoke exposure and obstructive airway disease in the study subjects

**Variable**	**Cases (%) (n=83)**	**Controls (%) (n=72)**	**P-value**
BHBS (yes/no)	43 (51.8)	22 (30.6)	<0.01
Frequency of BHBS (per week)*	4.87 (4.40)	2.28 (1.27)	0.01
No exposure	40 (48.2)	50 (69.4)	
Once	6 (7.2)	4 (5.6)	
Twice	10 (12)	12 (16.7)	<0.01^*^
>3 times	27 (32.5)	6 (8.3)	
Duration of BHBS (years)*	20.46 (11.60)	15.38 (13.20)	0.02
No exposure	40 (48.2)	50 (69.4)	
0–10 years	12 (14.5)	15 (20.8)	
11-20 years	15 (18.1)	3 (4.2)	<0.01^*^
>20 years	16 (19.3)	4 (5.6)	

^*^Data for the frequency of BHBS and duration of BHBS are mean (SD).

^*^According to Chi square for trend test.

According to the point estimate adjusted odds ratio of 2.22 and the prevalence of indoor exposure to BHBS of 51.8% in the cases, the PAF was approximately measured as 28.5% i.e. 28.5% of the OAD in the population is due to BHBS exposure.

## Discussion

The results of present study showed that there is a positive association between indoor exposure to BHBS and OAD which is consistent with the previous studies [15-18]. In an Iranian case-series by Amoli, all the women suffering from COPD were non-cigarette smokers and did not have a history of occupational exposure to dust; however, all reported a history of previous exposure to smoke during baking home-made bread for a long period [12]. Saha *et al.* showed that the use of solid fuels (especially wood) is an important factor in destruction of lung function and the individuals using these materials have a poorer pulmonary function compared with those consuming liquid petroleum gas [19].

More than two fold increase in risk of OAD in the subjects with a history of indoor exposure to BHBS in this study is consistent with the results of the meta-analysis by Kurmi *et al.*[20]. This study showed that exposure to the solid fuel smoke is strongly associated with chronic bronchitis (CB) and COPD, regardless of fuel type; solid fuel use increased the overall risk more than two times. In their study, exposure to wood smoke, among the solid fuel, was recognized as the major risk factor for COPD (odds ratio = 4.29).

In present study it was also found that more than 25% percent of the cases of OAD in the study population are attributable to BHBS exposure. These findings are compatible with the study by Ekici *et al.*[21] which indicated that, after controlling for the confounders, almost 23% of the COPD cases were attributable to exposure to smoke for a long period. Dennis et al. [15] estimated the PAF of wood smoke exposure for patients with OAD to be approximately 50%; this inconsistency could be justified by not considering the role of confounders in measuring the PAF in their study.

There is a big knowledge gap in the dose–response relationship between exposure to the solid fuel smoke and COPD and there are limited studies considering the hazardous role of the IAP in developing COPD. Kurmi *et al.*[20] in a systematic review considering the relationship between use of solid fuels and COPD, expressed that among the 23 investigations, none of the studies expressed any information about the dose–response relationship between the exposure and the disease. However, the findings of this study provided some evidences for dose–response relationship between indoor exposure to BHBS and OAD. Increased duration (years) and frequency (per week) of indoor exposure to BHBS increased the risk of developing OAD which is consistent with the results Dennis et al. [15].

IAP is a major bio-environmental problem in the rural areas of Iran which is due to the use of wood, animal dung, agricultural crop residues and coal for heating, cooking food and baking bread. Although there has been a decrease in use of solid fuels and wood for baking home-made bread in recent years, however, in many rural areas of our country using solid fuels and wood for baking home-made bread in ground ovens is still a common practice; this is done during the cold seasons within closed places with inadequate ventilation resulting in increased smoke exposure. Hence, indoor exposure to BHBS is considered a major hazardous bio-environmental problem for health in our country [22].

The current study showed that the duration of indoor exposure to BHBS (years) and the frequency of indoor exposure to BHBS (per week) was significantly higher in the subjects with OAD compared with the control group; this is similar to the findings of Akhtar *et al.*[16] who showed that the women spending two hours a week cooking for more than 10 years are at a higher risk for CB.

This study showed no relationship between smoking, occupational exposure to dust and low education level with OAD. Lack of significant relationship between OAD and occupational exposure to dust in this study is inconsistent with previous findings [23]. Denis *et al.* reported a significant association between cigarette smoking and OAD [15]. Previous reports about the etiology of COPD in Iranian women showed that the disease often occurs among the non-smokers without occupational exposure to dust who mention a history of prolonged exposure to smoke during baking bread in the past [12]. Liu and colleagues showed that there is no association between COPD and occupational exposure to dust or low education level among the non-smoker women [17].

The relationship between indoor exposure to BHBS and OAD is controversial in the two genders; there is a majority of studies expressing that the use of solid fuels is responsible for COPD among the non-smoker women living in rural areas of the developing countries [24,25]. However, in this study, we did not observe transgender difference regarding indoor exposure to BHBS and OAD occurrence which is consistent with the results of the study by Saha *et al.*[19]. Pandey showed in 1984 that in the rural areas of Nepal, where solid fuels are mostly used for cooking and heating, the prevalence of CB and COPD was similar in both genders [26].

In recent years in Iran, simultaneous use of multiple fuels and rapid change from one to another fuel in order to bake bread is a common practice. As a result, in our study we were not able to determine the type of solid fuel used; hence, only indoor exposure to BHBS was considered as the main variable. Also, as another limitation of this study, we did not consider passive smoking as a confounder.

## Competing interests

The authors declare that they have no competing interests.

## Authors' contributions

Both MQ and MY 1) have made substantial contributions to conception and design, or acquisition of data, or analysis and interpretation of data; 2) have been involved in drafting the manuscript or revising it critically; and 3) read and approved the final manuscript.
